# Effects of a Gamified, Behavior Change Technique–Based Mobile App on Increasing Physical Activity and Reducing Anxiety in Adults With Autism Spectrum Disorder: Feasibility Randomized Controlled Trial

**DOI:** 10.2196/35701

**Published:** 2022-07-28

**Authors:** Daehyoung Lee, Georgia C Frey, Donetta J Cothran, Jaroslaw Harezlak, Patrick C Shih

**Affiliations:** 1 Department of Applied Human Sciences University of Minnesota Duluth Duluth, MN United States; 2 Department of Kinesiology Indiana University Bloomington, IN United States; 3 Department of Epidemiology and Biostatistics Indiana University Bloomington, IN United States; 4 Department of Informatics Indiana University Bloomington, IN United States

**Keywords:** gamification, behavior change techniques, physical activity, sedentary behavior, anxiety, autism, mobile app, mental health, mHealth, mobile phone

## Abstract

**Background:**

Physical activity (PA) has an impact on physical and mental health in neurotypical populations, and addressing these variables may improve the prevalent burden of anxiety in adults with autism spectrum disorder (ASD). Gamified mobile apps using behavior change techniques present a promising way of increasing PA and reducing sedentary time, thus reducing anxiety in adults with ASD.

**Objective:**

This study aimed to compare the effectiveness of a gamified and behavior change technique–based mobile app, PuzzleWalk, versus a commercially available app, Google Fit, on increasing PA and reducing sedentary time as an adjunct anxiety treatment for this population.

**Methods:**

A total of 24 adults with ASD were assigned to either the PuzzleWalk or Google Fit group for 5 weeks using a covariate-adaptive randomization design. PA and anxiety were assessed over 7 days at 3 different data collection periods (ie, baseline, intervention start, and intervention end) using triaxial accelerometers and the Beck Anxiety Inventory. Group differences in outcome variables were assessed using repeated-measures analysis of covariance, adjusting for age, sex, and BMI.

**Results:**

The findings indicated that the PuzzleWalk group spent a significantly longer amount of time on app use compared with the Google Fit group (*F*_2,38_=5.07; *P*=.01; partial *η*^2^=0.21), whereas anxiety was unfavorably associated with increases in light PA and decreases in sedentary time after intervention (all *P*<.05).

**Conclusions:**

Further research is needed to clarify the determinants of physical and mental health and their interrelationship in adults with ASD to identify the factors that facilitate the use and adoption of mobile health technologies in these individuals. Despite these mixed results, the small changes in PA or anxiety may be clinically significant for adults with ASD.

**Trial Registration:**

ClinicalTrials.gov NCT05466617; https://clinicaltrials.gov/show/NCT05466617

## Introduction

Regular physical activity (PA) helps to reduce anxiety in the neurotypical population [1,2] and presents a potentially effective adjunct treatment for anxiety in people with autism spectrum disorder (ASD). Anxiety is one of the most common and debilitating mental health issues among adults with ASD [3,4]. A recent systematic review and meta-analysis study revealed that the lifetime prevalence of anxiety was >40% in a large sample of adults with ASD (n=26,070) included in the study [4]. The negative impact of chronic anxiety on those with ASD has been well documented, and evidence suggests that the presence of comorbid psychiatric disorders significantly interferes with active daily living and further increases the risk of clinical morbidity in these individuals [5,6].

Despite the prevalence and negative impact of anxiety on the everyday lives of those with ASD [7], there are few effective treatment options for this symptom. Most people with ASD rely on medications, which carry a high dependency risk [8,9], or cognitive behavioral therapy, which has demonstrated mixed results in alleviating this symptom [10,11]. In addition, antianxiety medication use is associated with several side effects, such as metabolic syndrome and weight gain [12,13], whereas cognitive behavioral therapy is both costly and labor intensive [14,15]. As a result, efforts have been made to identify adjunct treatments to help adults with ASD better manage anxiety [16,17]. The adult population with ASD is increasing [18,19], and there is a critical need for accessible and cost-effective adjunct anxiety treatments to alleviate this problem.

Although the level of benefits may vary from person to person, participation in regular PA is proven to help alleviate anxiety symptoms by improving self-efficacy [20], strengthening the sympathetic nervous system [21], and increasing neurogenesis in the human brain [22]. Research on PA interventions for adults with ASD is markedly lacking; however, there is support for the positive impact of PA or exercise on mental health in this population. Hillier et al [23] found that salivary cortisol and self-reported anxiety were reduced immediately after a low-intensity exercise session 1 day per week in a small sample of young adults with ASD. These effects were not maintained over the course of the 8-week intervention; however, the findings suggest that even low-intensity exercise can have a positive, albeit short-term, effect on anxiety in these individuals [23]. It is of interest to determine whether participation in regular PA can induce similar results in adults with ASD in the long term.

Although it is well understood that PA is a leading health indicator for both physical and mental health [24-26], there is little information on PA participation in adults with ASD. To date, only a few studies have addressed PA levels in adults with ASD, and the results have varied greatly. Eaves and Ho [27] reported that adults with ASD only engaged in moderate to vigorous PA (MVPA) once per week and spent approximately 13 hours per day sitting. In contrast, Frey et al [28] and Garcia-Pastor et al [29] found that adults with ASD met the guidelines of 150 minutes of weekly MVPA but were also highly sedentary. Lalonde et al [30] also observed that adults with ASD had daily step counts similar to neurotypical adults. It is difficult to draw clear conclusions about these findings because of variability in the assessment methods and the functional ability of the samples. Most of the previous study findings were based on participants who attended segregated schools or centers and required extensive support, which was not representative of adults with ASD who were more intellectually able and autonomous.

To date, a limited number of studies have specifically attempted to increase PA in adults with ASD by using objective measures. Lalonde et al [30] used a goal-setting–based and reinforcement-based treatment to increase pedometer-measured walking steps in 5 young adults with ASD who attended a special education program. All participants were able to increase the number of walking steps to sufficient levels to achieve health benefits (ie, ≥10,000 steps). Although these are encouraging findings, the highly structured and prompted nature of the intervention makes it inappropriate to generalize the findings to adults with ASD who are more intellectually able and autonomous. There has also been an effort to address physical fitness in individuals with ASD using a video modeling–incorporated mobile app. Although this novel intervention approach is promising, as the app was effective in increasing the heart rate and energy expenditure of participants with ASD, the findings were limited to children with ASD who required substantial assistance and supervision for proper execution [31]. It is important to identify PA and sedentary time interventions that are more suitable for independent adults with ASD who are self-determined for health behaviors.

Technology-based PA interventions have proven to be a promising method of increasing PA in the neurotypical population [32-34] and may be well suited for adults with ASD. Gamified mobile technology is a particularly promising tool that can increase PA and reduce sedentary time while also meeting the unique needs and interests of these individuals [35-37]. Lee et al [37] found that adults with ASD used technological devices for >6 hours per day, primarily for surfing the internet, using social media, and web-based gaming [37]. Emerging evidence suggests that adults with ASD are attracted to technology use because the human-technology interface creates consistency and predictability, as well as because of a lower social burden, compared with traditional face-to-face interaction [38,39]. Furthermore, individuals with ASD typically have distinctive strengths in visuospatial functioning, which is common to technology-based games, as well as a preference for learning and interaction through visual information [40-42]. Consequently, in the past few decades, health practitioners have actively used mobile technologies to offer a cost-effective and low-barrier platform for visual learning to identify and improve health outcomes in diverse clinical populations, including those with ASD [37,38,43].

Gamified behavioral interventions using smartphone apps have the advantage of providing personalization, feelings of amusement, and desire for continuation [44-46] and have rapidly expanded their technological potential to monitor and improve daily PA participation in adults with obesity and sedentary workers [35,47]. Nevertheless, the success of gamified mobile apps in promoting PA and reducing sedentary behavior is questionable as most of the existing health or fitness apps in the commercial market are not sustainable stand-alone interventions and lack scientific evidence and health behavior theory in the app development process [48,49]. The overall low quality of evidence regarding the effectiveness of long-term behavior change makes it currently difficult to apply commercial PA-promoting apps to adults with ASD [50]. The purpose of this study was to (1) examine the effects of the competitive gamification and behavior change theory–based mobile app PuzzleWalk [37] on increasing PA and reducing sedentary time and anxiety in adults with ASD and (2) compare PuzzleWalk to a commercially available platform, Google Fit. It is hypothesized that (1) the use of PuzzleWalk will lead to higher levels of light PA and MVPA and lower levels of sedentary time and anxiety in adults with ASD than the use of Google Fit and (2) the increased PA or decreased sedentary time from both apps will be associated with reduced levels of anxiety in adults with ASD.

## Methods

### Participants

A total of 29 adults aged ≥18 years and diagnosed with ASD were recruited through state and regional agencies that serve people with ASD across the United States and online autism support groups on social media such as Facebook and Reddit. Evidence of ASD diagnosed by a qualified medical professional such as a pediatrician or clinical psychologist (ie, when and where) was required for study participation and obtained via self-report. In addition, eligible study participants met the following inclusion criteria: (1) self-reported medical diagnosis of anxiety or self-identification of experiencing anxiety symptoms for the past 3 or more months, (2) access to a supported device (smartphones with Android 4.4 and higher or iOS 9.0 and higher operating system), (3) cognitive ability to understand the purpose of the study, and (4) no prior experience using the PA mobile apps used in the study. Individuals with low cognitive function, co-occurring intellectual disabilities, or mobility impairments were excluded from this study. We hereby use the term *intellectually able* adults with ASD to refer to those who can make their own decisions regarding health behaviors without much assistance. A participant with a self-reported mild learning disorder that did not significantly interfere with active daily living and study participation was included in the study. A formal screening interview was conducted with each participant through a phone or face-to-face video call to verify participant eligibility and identify potential barriers to study participation. All participants provided written or digital consent before data collection.

### Ethics Approval

The Institutional Review Board of Indiana University approved this study (protocol number 1807483245).

### Procedure

On verification of the participant’s eligibility through the screening interview, participants completed a web-based demographic survey with an emphasis on BMI, waist circumference, medication use, and autism symptoms. Self-reported height and weight information were collected to calculate each participant’s BMI (ie, kg/m^2^ or lbs/in^2^ × 703) [51]. Participants were also asked to provide self-measured waist circumference, which can be an indicator of obesity-related disease risk [52]. The Autism Spectrum Quotient 10-item (AQ-10) questionnaire was included in the survey to examine the severity of autism symptoms (eg, social interaction deficits, sensory issues, and other autism-specific characteristics) [53]. The AQ-10 was designed for health care professionals to administer an informal ASD assessment of individuals with typical cognitive function [54]. The maximum possible score is 10, and individuals with a score of ≥6 are advised to consider referral for formal ASD assessment. The AQ-10 is one of the few autism screening tools available for adults [18]. After the completion of the demographic survey, study materials, including an accelerometer with an elastic belt, a USB cable charger, and study instruction sheets (ie, how to wear and charge the accelerometer and how to install and use the daily anxiety assessment [DAA] app for anxiety assessment) were either mailed or handed to remote and local participants, respectively.

A rubric was used to assess participant comfort and knowledge of study procedures before the start of the baseline and intervention periods, and case-by-case decisions were made by the research team when individuals were ready to start data collection. Before the start of the intervention period, participants were assigned to either the PuzzleWalk or Google Fit group according to age, sex, and BMI using a covariate-adaptive randomization process. Specifically, a minimization technique was applied to the randomization process by distributing the participants into 2 groups based on the aforementioned variables, which were identified before the start of data collection [55]. Covariate randomization aimed to minimize the imbalance in baseline characteristics across the 2 groups included in the study [56]. All participants received visualized step-by-step instructions (eg, search and download on Google Play or App Store, user registration, goal setting, and PA behavior tracking) on the assigned PA app (PuzzleWalk or Google Fit) and used it from the beginning of the intervention start (fourth week) until the end of the intervention (eighth week).

**Figure 1:**
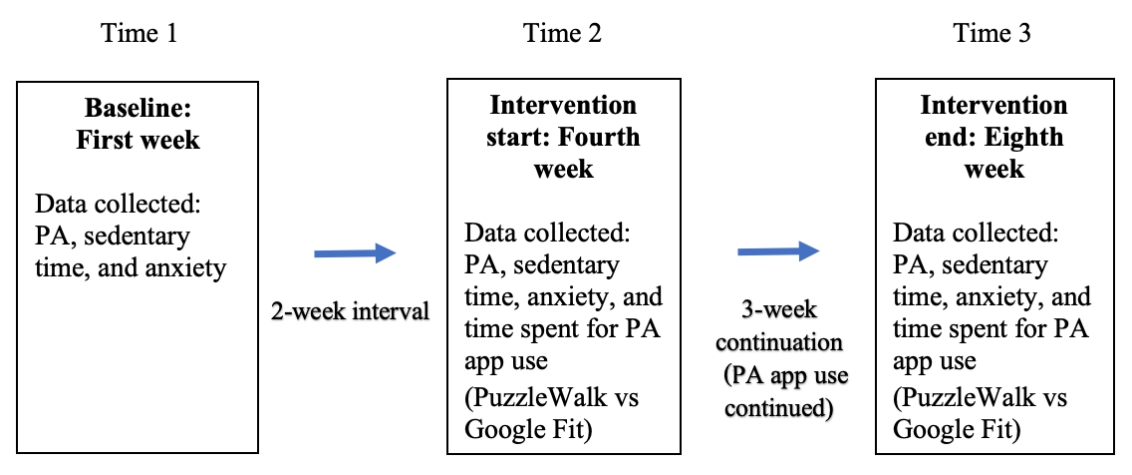
Data collection time points. PA: physical activity.

Participation required an approximate 2-month commitment: the first week for baseline and the fourth to eighth weeks for intervention. A 2-week interval was implemented between baseline and intervention onset to reduce the novelty effect in response to the accelerometer and PA app use [57] (see Figure 1 for the study timeline). Both the PuzzleWalk and Google Fit groups received reminders regarding the use of the PA app during the first week of the intervention period and autonomously continued to use the app until the intervention ended. Data collection occurred in the fall and early spring to avoid the impact of inclement weather on activity patterns. Participants who successfully completed the 2-month study received a US $100 e-gift card as a token of appreciation.

### Mobile Apps

#### Google Fit

Google Fit (Google LLC) is a PA-tracking platform developed by Google for Android and Apple iOS. The app was first launched to the public in 2014, and since its release, it has been one of the most popular health/fitness apps in the United States, with >2.6 million monthly active users (Statista 2022 [58]). The app uses a sensor built into a smartphone device to automatically track PA, including steps and active minutes. It also allows users to journal and record a variety of forms of PA (eg, cycling, weightlifting, and yoga) by manually setting the activity tracking mode. Google Fit uses a heart point–based reward system as a gamification strategy to provide users with individualized exercise tips incorporated with PA recommendations outlined by the American Heart Association. Google Fit users can earn one heart point for each minute of MVPA, with possible virtual rewards (ie, celebrative animation on the interface in addition to a green circle morphed into the user’s profile image) when they reach a certain number of PA milestones (eg, 30 minutes of moderate PA a day or 150 minutes of MVPA per week). The number of heart points received based on active minutes is the app’s primary gamification strategy. To the best of our knowledge, there are no peer-reviewed, data-based publications on the app’s functional reliability and behavior change effectiveness. There are a few industrial reports and studies on Google Fit, although these studies focused on the basic functionality of the app and compatibility with wearable trackers [59,60]. We chose Google Fit as a comparison platform as it is free and easy to use, and, most importantly, it has been extensively used by neurotypical adults. As such, the comparison of Google Fit in this study will provide valuable insights into the usability/feasibility of a commercially available health app for promoting PA in adults with ASD.

#### PuzzleWalk

A gamified behavior change app, PuzzleWalk, available for both Android and Apple iOS, was developed to increase PA and reduce sedentary behavior in adults with ASD following a participatory, user-centered development process, including a needs analysis, literature review, and prototype design [37]. PuzzleWalk incorporates behavior change techniques (BCTs), a theory-based method of promoting healthy behavior change by leveraging psychological determinants, such as autonomy, perceived competence, and intrinsic and extrinsic motivation [61]. The example techniques included in PuzzleWalk are a comprehensive, visualized user guide, self-monitoring of target performance, contingent rewards, and goal setting [37].

**Figure 2:**
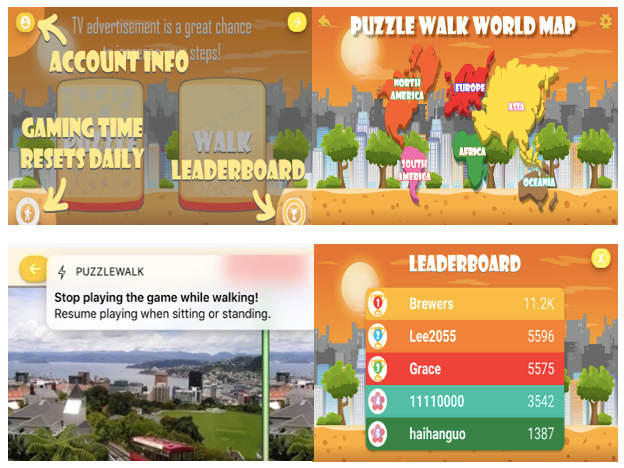
PuzzleWalk user interfaces.

It is a *spot the difference* puzzle game comprising 660 major city images around the world (see Figure 2). This format was chosen because it is easy to understand the purpose of the game, and it can quickly capture the user’s interests without a complex comprehension process. Moreover, this visual image–based game facilitates visual interaction, which is a unique strength of individuals with ASD [40]. The most unique design element of PuzzleWalk is the conversion algorithm between steps and game-solving time. Specifically, the user’s accumulated steps are directly converted to game-solving time to motivate PA participation. A review of the literature indicated that only a few available PA apps use this gamified token economy strategy for PA promotion. Pokémon Go uses a similar gamification strategy as the app links walking activities to the Pokémon character–hunting game supported by location tracking and augmented reality technologies; however, there is no direct conversion algorithm between PA (steps) and game time/opportunity. PuzzleWalk also uses a gamified leaderboard that ranks active users based on their steps and puzzle scores, with tangible rewards (ie, US $10 e-gift cards) provided to the top 3 score leaders at the end of each month. This gamified leaderboard leveraged a BCT of prompt rewards contingent on efforts toward a target behavior [62,63].

### Mobile App Use Assessment

PA app use for the past 1 week was assessed through a self-report survey at 3 different data collection time points (ie, end of the fourth week, start of the eighth week, and end of the eighth week) (see Table 1). Participants were asked to report the frequency (days of app use during the past 7 days) and duration (hours and minutes usually spent for app use). The time spent using the PA app was calculated using a minutes per day format.

**Table 1:** Durations of data collection.

Instrument used	Time 1: baseline (first week)	Time 2: intervention start (fourth week)	Time 3: intervention end (eighth week)	Total
Accelerometry (days)	7	7	7	21
Beck Anxiety Inventory for the past 7-day anxiety assessment	Days 1 and 7	Days 1 and 7	Days 1 and 7	6 times
Daily anxiety assessment (days)	7	7	7	21
Survey for physical activity app use	N/A^a^	Day 7	Days 1 and 7	3 times

^a^N/A: not applicable.

### Anxiety Assessment

The Beck Anxiety Inventory (BAI) was used to assess participants’ prolonged state of anxiety. The BAI is a self-report scale comprising 21 items that measure the severity of anxiety symptoms during the past week, with high internal consistency (Cronbach α=.92) and test-retest reliability (*r*=0.75) [64]. Scores range from 0 to 63, with scores of ≥26 indicating potentially concerning levels of anxiety (ie, 0-7=minimal; 8-15=mild; 16-25=moderate, and ≥26=concerningly severe). The participants were asked to report the extent to which they had been bothered by each of the 21 symptoms in the past week. The participants completed the BAI at the start and end of each data collection period (a total of 6 times).

In addition to the BAI, time-specific and type of anxiety trigger questions were asked daily during each data collection period to better identify the contexts of potential anxiety triggers such as environmental, psychological, or sensory factors [65]. At the start of the study, participants downloaded the DAA mobile app developed by the research team. The participants received a prompt at 8 PM each day during the data collection period to answer specific questions. This time was deemed appropriate for assessing perceived emotional changes over the course of the day based on pilot work and considering typical work/school schedules and bedtimes. The questions included “When did you feel anxious today?” and “What caused your anxiety today?” with the possible choices of (1) change or disruption to routine, (2) sensory oversensitivity or overstimulation, (3) confusion and worries about social and communication situations, (4) specific fears or phobias, (5) too many demands or expectations, (6) being prevented from preferred behaviors or interests, and (7) not applicable. As the traditional retrospective assessment approach can be less reliable [66], the DAA adopted the ecologic momentary assessment method to improve response reliability [67]. Low compliance can be an issue with this type of daily report; therefore, we implemented routine strategies to address this issue, including establishing an efficient data/compliance-tracking system and providing regular reminders to complete the task and monetary incentives [68]. Reminders were sent to the participants via email or SMS text messages based on their preferences recorded in the demographic survey. Only participants with valid survey compliance (ie, participation for 6 times in the BAI and participation in at least four DAAs in each data collection period) and monitor wear (ie, at least 3 valid days in each data collection period) received a monetary incentive.

### PA and Sedentary Time Assessment

Daily walking steps, PA intensity, and sedentary time were measured using GT3X+ and ActiGraph triaxial accelerometers (ActiGraph). ActiGraph accelerometers have been extensively used to measure PA with moderate to high reliability in both laboratory and free-living conditions [69-71]. All participants were asked to wear an accelerometer on their right hip during waking hours for 7 consecutive days, including at least 2 weekdays and 1 weekend day over the 3 different data collection periods (baseline, start of the fourth-week intervention, and end of the eighth-week intervention). Accelerometers were programmed to calculate data in 60-second epochs [72]. Daily walking steps were measured using the ActiGraph pedometer function, and sedentary time and activity intensity were identified using the following activity counts per minute cutoffs: <100=sedentary, 100-2019=light, 2020-5999=moderate, >5999=vigorous, and ≥2020=MVPA [73]. These cutoff points were proposed by Troiano et al [73] and have been validated in large data sets of free-living adults [74-76]. The minimum wear time for a valid day is ≥10 hours per day for wake time [77]. Although there is no scientific consensus on the minimum number of valid days, 4 days have been widely accepted in previous studies to reliably estimate habitual PA [78,79]. However, this study required at least 3 valid days of monitor wear in each data collection period in an effort to minimize sample loss [78].

In addition, an intraclass correlation (ICC) analysis was performed to validate the use of at least 3 valid days of monitor wear by examining the relationship between 3 valid days and ≥4 valid days on all PA and sedentary time outcomes at baseline. ICC values of ≥0.75 are generally regarded as good or acceptable reliability [80]. The ICC results demonstrated that the reliability of 3 valid days for all PA and sedentary time variables was excellent (all ICC >0.90); therefore, participants with ≥3 valid days of monitor wear were included in the analyses. Overall, 2 participants’ accelerometry data at the intervention start (fourth week) and 1 participant’s accelerometry data at the intervention end (eighth week) did not satisfy the minimum number of 3 valid days; thus, their baseline accelerometry data were imputed according to the baseline observation carried forward method [81]. The criterion for nonwear time was 90 minutes of consecutive 0 counts [82], and the accelerometers were set to collect data at sampling rate of 30 Hz.

### Data Analysis

Independent *t* tests and chi-square tests were performed for continuous and ordinal variables, respectively, to compare the baseline differences between the 2 groups. Data are presented as mean (SD) or frequency (percentage), according to the variable type. According to the scoring guidelines, the BAI scores for all 21 items (ie, not at all=0, mild=1, moderate=2, and severe=3) were summed to yield a total score of anxiety severity [64]. The BAI scores collected twice during each data collection period were averaged to represent each time point (ie, baseline, intervention start, and intervention end). Data on common anxiety triggers and time-specific occurrences were screened to include only participants with at least four responses to the DAA at each data collection time point. All participants met the compliance criteria, and 134 counts (baseline, N=24), 124 counts (intervention start, 23/24, 96%), and 107 counts (intervention end, 20/24, 83%) were included in the frequency (percentage) analyses.

Objectively measured PA data were first systematically cleaned and verified by examining the ranges and missing values according to the established validation criteria. The collected raw accelerometry data were then converted into activity counts using the ActiLife 6 Data Analysis Software (ActiGraph). With the aforementioned activity intensity cutoffs, the PA data were processed and extracted into an editable spreadsheet. Manual data screening was sequentially performed to verify the minimum required hours and valid days of monitor wear for data analysis; invalid data were eliminated.

An intention-to-treat analysis was conducted to maximize external validity, and the baseline observation carried forward method was used to impute missing data after randomization [81,83,84]. According to the guidelines on missing data in clinical trials by the European Medicines Agency (2010), the baseline observation carried forward method can be appropriate in randomized trial design studies in which researchers reasonably assume that the outcomes of a participant would return to their baseline levels in the long term after dropout [85]. In light of the general span of a mobile health intervention’s effectiveness in promoting PA (eg, up to 3 months) [86], this method can also help maintain external validity and minimize sample loss.

The dependent variables were sedentary time (minutes per day), light PA (minutes per day), MVPA (minutes per day), steps per day, total activity counts (vector magnitude), average BAI anxiety score, and PA app use (minutes per day). To assess PA and anxiety changes over the 3 data collection periods; all measures collected at baseline, intervention start, and intervention end were compared between time points and groups using a repeated-measures analysis of covariance (ANCOVA). Repeated-measures ANCOVA models were adjusted for baseline characteristics, including age, sex, and BMI. The Mauchly test of sphericity was used for each outcome variable to examine the equality of variances of within-group differences across the 3 different data collection time points. In general, if the *P* value was <.05, the assumption of sphericity was violated. Owing to the violation of the sphericity assumption, the Greenhouse-Geisser correction was applied to MVPA, steps, and total activity count variables to interpret the results of the within-group effects.

The effect size (partial *η*^2^) was calculated and defined as >0.02=small, >0.13=medium, or >0.26=large [87]. Owing to the violation of the normality assumption, Spearman rank correlation analyses were performed to determine the baseline correlations between the outcome variables and the impact of increased PA or decreased sedentary time on anxiety change. The degree of change in the outcome variables following PA app use was calculated by subtracting the baseline value from the average of intervention outcomes (eg, MVPA change = [MVPA at intervention start + intervention end]/2 – MVPA at baseline). Data analyses were performed using SPSS 26.0, and significance was declared at *P*<.05 (2-tailed).

## Results

A total of 29 adults with ASD initially volunteered to participate in this study. Approximately 17% (5/29) of participants were eliminated before the start of data collection as they did not meet the eligibility criteria or lost study materials. The remaining 83% (24/29) of participants met the eligibility criteria and were enrolled in the study. Of the 24 participants, 3 (13%) from the PuzzleWalk group and 1 (4%) participant from the Google Fit group dropped out during either the intervention start or intervention end time point because of personal obligation (n=1, 4%), invalid monitor wear compliance (n=1, 4%), and restrictions on outdoor activities because of the COVID-19 pandemic (n=2, 8%). The retention rate was 83.3%. On the basis of the intention-to-treat standard, the baseline observation carried forward method was applied to these 4 cases; thus, no participants were lost after data collection was started. Overall, there were no statistically significant baseline differences between the 2 groups. The participant characteristics are presented in Table 2.

**Figure 3:**
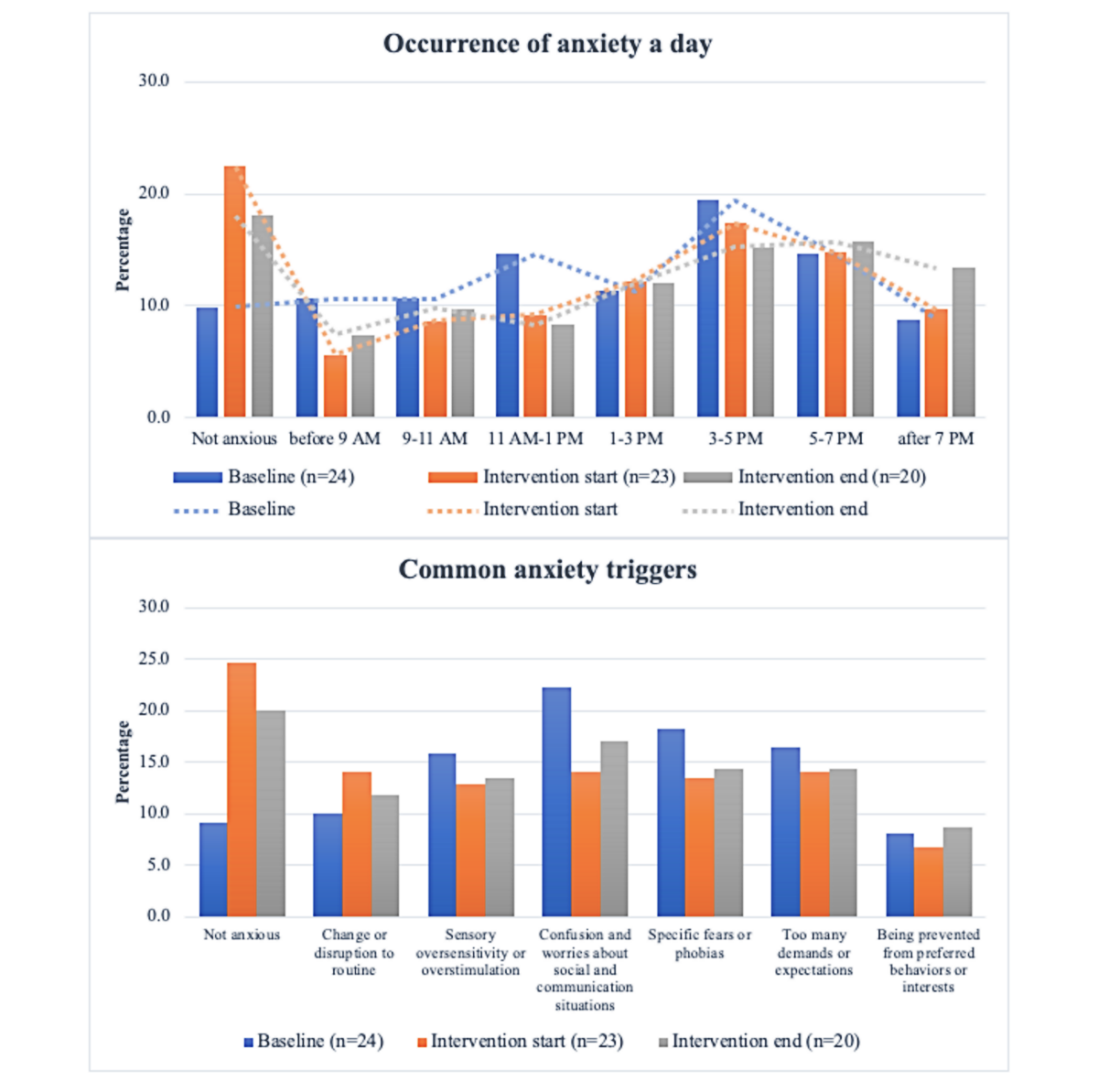
Time-specific occurrence of anxiety and perceived anxiety triggers from baseline to intervention end. Multiple answers were allowed for both questions. At least four responses to the Daily Anxiety Assessment were required for each 7-day data collection period. The loss of 4 participants during the intervention period was accounted for in the percentage calculation.

The average valid monitor wear for baseline, start of the intervention, and end of the intervention were for 5.8 (82.9%; SD 1.6) days, 5.7 (81.4%; SD 1.6) days, and 5.6 (80.0%; SD 1.4) days, respectively. Of the 24 participants, 13 (54%) at baseline, 10 (46%) at the start of the intervention, and 7 (35%) at the end of the intervention = wore the monitor for the full 7 days. On average, participants wore the monitor for 14.4 (SD 1.7) hours per day, 14.3 (SD 2.1) hours per day, and 14.0 (SD 2.0) hours per day during each data collection period. Regarding anxiety occurrence, adults with ASD experienced anxiety more frequently during the late afternoon—between 3 PM and 7 PM. Overall, participants felt relatively less anxiety during the week of intervention start; however, this positive change was slightly diminished during the last week of intervention (Figure 3).

**Figure 4:**
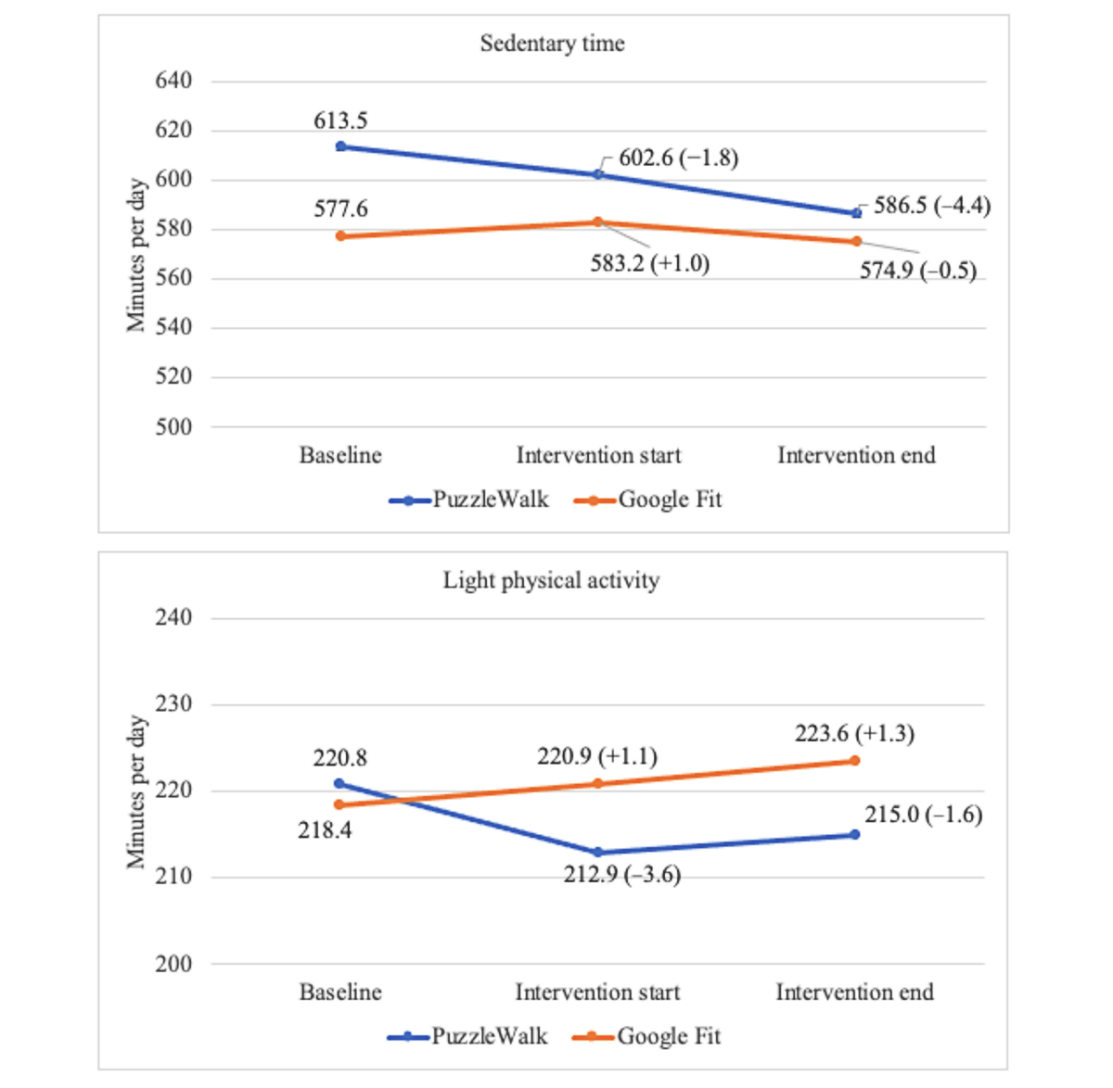
Between- and within-group comparisons of sedentary time and light physical activity. Data are presented as mean (percentage change from baseline).

**Figure 5:**
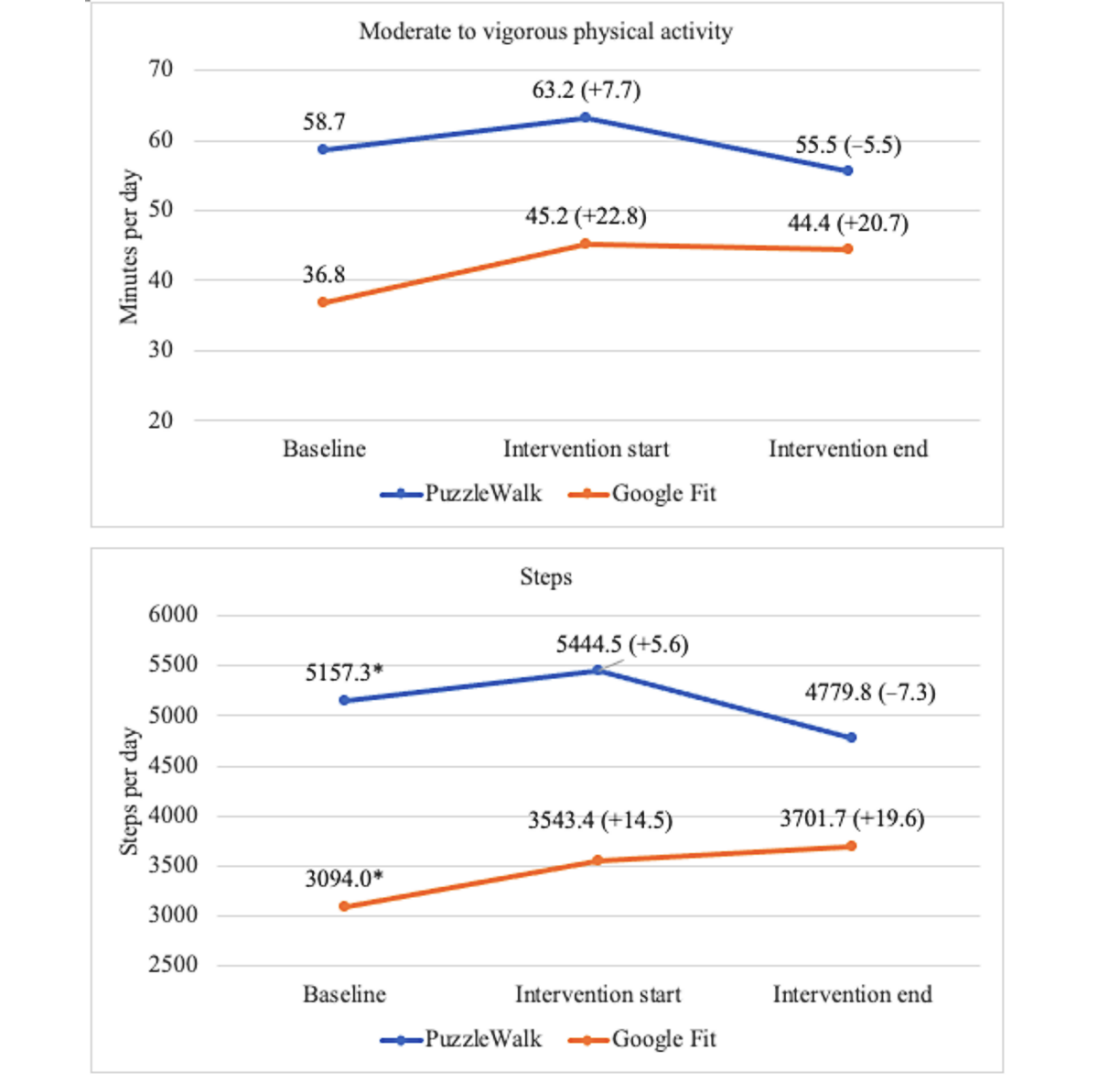
Between- and within-group comparisons of moderate to vigorous physical activity and steps. Data are presented as mean (percentage change from baseline). *P<.05, between-group difference.

**Figure 6:**
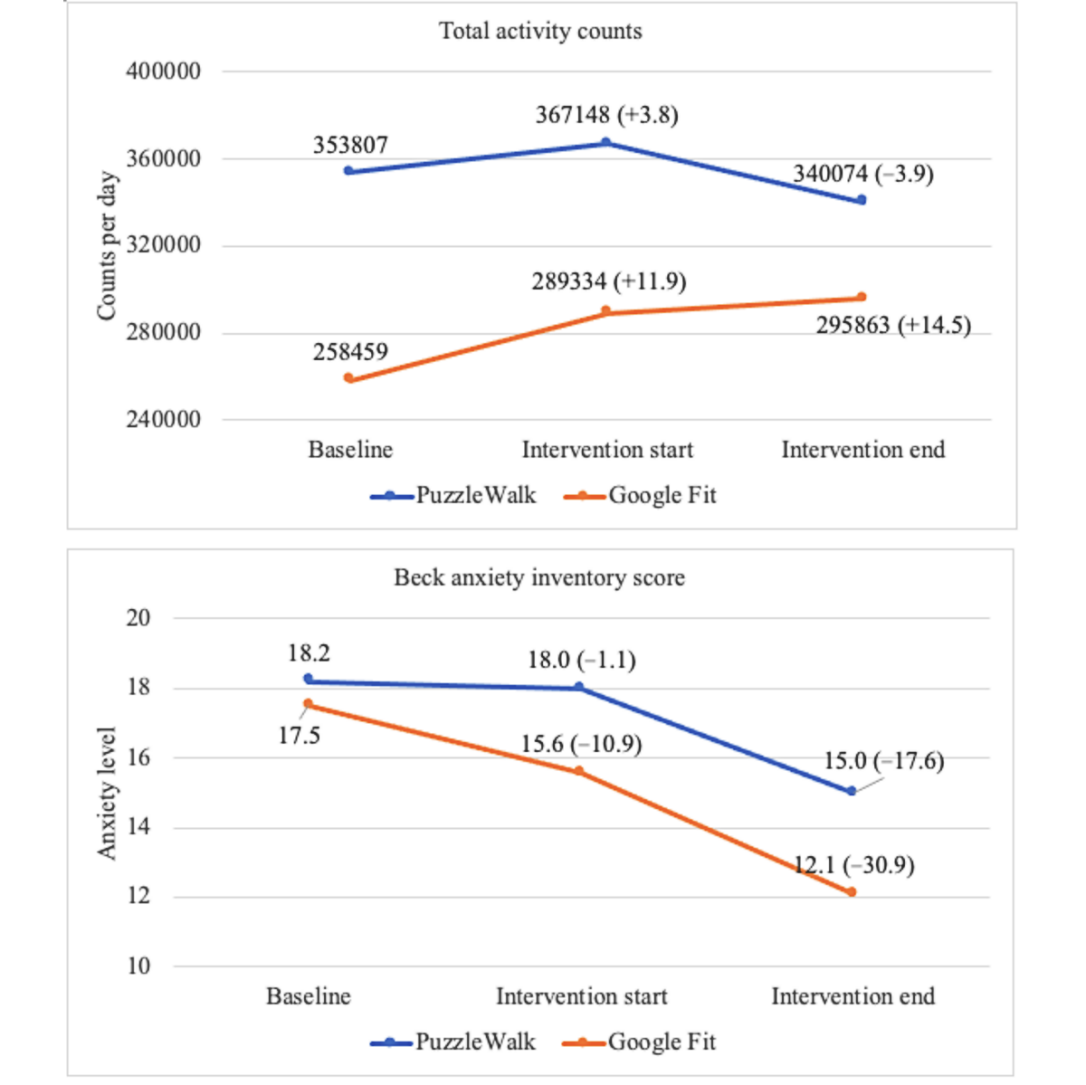
Between- and within-group comparisons of total activity counts and anxiety level. Data are presented as mean (percentage change from baseline).

**Figure 7:**
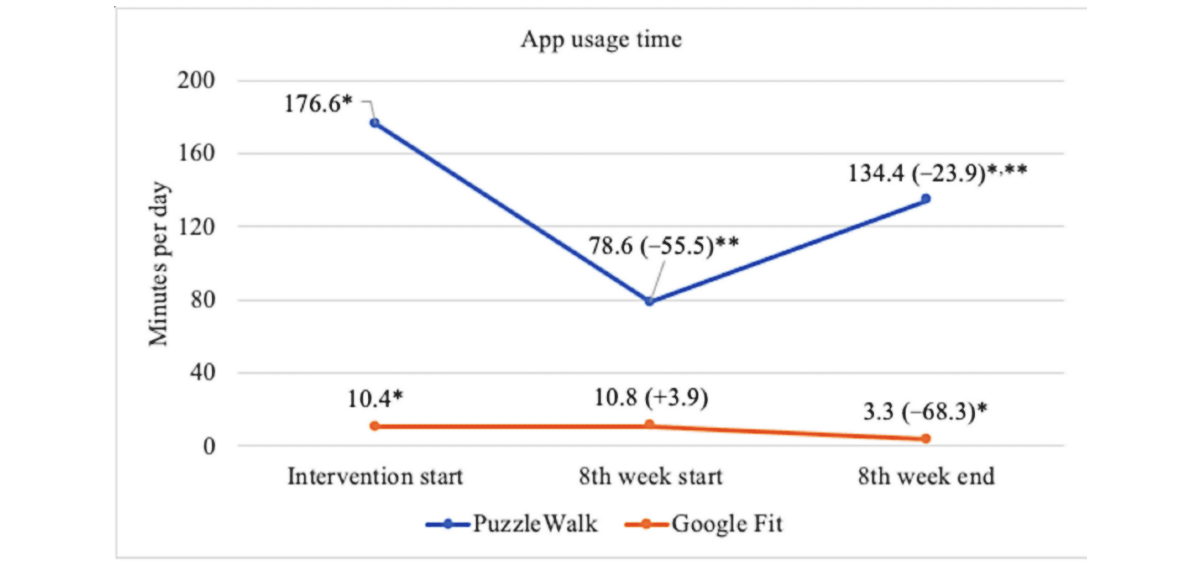
Between- and within-group comparisons of app usage time. Data are presented as mean (percentage change from baseline). *P<.05, between-group difference; **P<.05, within-group difference.

Figures 4-7 show the descriptive statistics (mean and percentage change) for all outcome measures, including sedentary time, light PA, MVPA, steps, total activity counts, BAI score, and time spent on app use across the 3 data collection periods between the PuzzleWalk and Google Fit groups. The only baseline difference was in daily steps (PuzzleWalk mean 5157.3, SD 2987.2 steps per day, vs Google Fit mean 3094.0, SD 1506.1 steps per day; *P*=.04). There were no significant changes in any of the PA or sedentary time variables over time in either group. The app use time was significantly different between the 2 groups at intervention start (*P*=.046) and intervention end (*P*=.045). The PuzzleWalk group showed a significantly decreased time spent on app use at the start of the final intervention week (*P*=.04); however, the time increased at the end of the intervention (*P*=.049).

Repeated-measures ANCOVAs were performed with age, sex, and BMI as covariates to test if there was a time×group interaction effect between the 2 groups (Tables 3 and 4). There was a significant within-group change over time in sedentary time (*P*=.003) and MVPA (*P*=.04). PA app use was the only variable that resulted in statistically significant pairwise and overall between-group and time×group interaction differences. Specifically, PuzzleWalk participants showed a significant decrease in time spent on PA app use from the start of the intervention to the start of the eighth week (mean 203.5, SD 62.6 minutes per day, to mean 82.9, 38.2 minutes per day; *P*=.008); however, time significantly increased from the start of the eighth week to the end of the intervention (mean 82.9, SD 38.2 minutes per day, to 162.9, 48.3 minutes per day; *P*=.01). In addition, pairwise group differences were found to be statistically significant between PuzzleWalk and Google Fit groups at the start of the intervention (mean 203.5, SD 62.6 minutes per day, vs 12.9, 57.7 minutes per day; *P*=.04) and at the end of the intervention (mean 162.9, SD 48.3 minutes per day, vs 3.3, SD 44.5 minutes per day; *P*=.03) periods. When it comes to overall time×group interactions, there were significant overall between-group (*P*=.04) and time×group interaction (*P*=.01) effects on the time spent on PA app use, indicating that the overall increase in PA app use time was considerably higher in the PuzzleWalk group than in the Google Fit group.

Sedentary time was significantly negatively associated with MVPA (*P*=.03), steps (*P*<.01), and total activity counts (*P*=.021), whereas MVPA was positively associated with steps (*P*<.01) and total activity counts (*P*<.01) at baseline, and these relationships remained significant after the intervention. Anxiety level was not significantly associated with any PA variables or sedentary time at baseline but was changed to have a significant negative association with sedentary time (*P*=.02) and positive associations with light PA (*P*=.045), steps (*P*=.03), and total activity counts (*P*=.045) after the intervention (Table 5).

**Table 2:** Baseline characteristics of study participants (N=24).

Characteristics			PuzzleWalk (n=12)		Google Fit (n=12)		Total		*P* value
Age (years), mean (SD)			27.1 (7.5)		31.9 (11.3)		29.5 (9.7)		.23
**Age at diagnosis (years), n (%)**									
	Early childhood (birth to age 5)	3 (25)		1 (8)		4 (17)		.74	
	Later childhood (age 6-11)	2 (17)		3 (25)		5 (21)		.74	
	Adolescence (age 12-17)	1 (8)		1 (8)		2 (8)		.74	
	Adulthood (age ≥18)	6 (50)		7 (58)		13 (54)		.74	
Female, n (%)			9 (75)		6 (50)		15 (63)		.40
Autism symptoms (AQ-10 score^a,b^), mean (SD)			7.9 (1.6)		8.6 (1.6)		8.3 (1.6)		.33
BMI, mean (SD)			32.2 (7.9)		30.3 (8.9)		31.3 (8.3)		.59
Waist circumference, mean (SD)			38.1 (5.5)		39.3 (5.7)		38.7 (5.5)		.63
**Education, n (%)**									
	High school or General Education Diploma	0 (0)		4 (33)		4 (17)		.16	
	Some college	5 (42)		4 (33)		9 (38)		.16	
	College degree	6 (50)		3 (25)		9 (38)		.16	
	Postgraduate	1 (8)		1 (8)		2 (8)		.16	
**Employment status, n (%)**									
	Unemployed	1 (8)		4 (33)		5 (21)		.44	
	Nonpaid work (eg, volunteer)	1 (8)		2 (17)		3 (13)		.44	
	Keeping house or homemaker	1 (8)		0 (0)		1 (4)		.44	
	Student	4 (33)		3 (25)		7 (29)		.44	
	Paid employment	5 (42)		3 (25)		8 (33)		.44	

^a^AQ-10: Autism Spectrum Quotient 10-item.

^b^With a possible maximum score of 10, a higher score on the AQ-10 indicates the presence of more autism symptoms.

**Table 3:** Summary of changes in PA^a^ and anxiety from baseline to intervention end^b^.

Variable			Baseline, mean (SE)		Intervention start, mean (SE)		Intervention end, mean (SE)		Time, *F* test (*η*_p_^2^; *df*=2,38)		Between-group, *F* test (*η*_p_^2^; *df*=2,38)		Time×group, *F* test (*η*_p_^2^; *df*=2,38)
**Sedentary time (minutes per day)**													
	PuzzleWalk	644.0 (31.0)		635.8 (36.2)		627.1 (43.9)		6.83 (0.26)^c^		1.96 (0.09)		0.13 (0.01)	
	Google Fit	567.5 (28.6)		572.9 (33.3)		557.3 (40.5)		6.83 (0.26)^c^		1.96 (0.09)		0.13 (0.01)	
**Light PA (minutes per day)**													
	PuzzleWalk	226.2 (17.5)		220.5 (20.7)		220.7 (19.5)		0.23 (0.01)		0.02 (0.00)		0.24 (0.01)	
	Google Fit	215.6 (16.1)		217.3 (19.0)		224.4 (18.0)		0.23 (0.01)		0.02 (0.00)		0.24 (0.01)	
**Moderate to vigorous PA (minutes per day)**													
	PuzzleWalk	55.9 (9.2)		61.3 (11.2)		50.8 (9.1)		3.47 (0.15)^c^		1.04 (0.05)		1.52 (0.07)	
	Google Fit	36.9 (8.5)		45.7 (10.3)		46.3 (8.4)		3.47 (0.15)^c^		1.04 (0.05)		1.52 (0.07)	
**Steps (steps per day)**													
	PuzzleWalk	4815.9 (787.1)		5083.0 (892.8)		4298.6 (779.2)		1.80 (0.09)		1.28 (0.06)		1.71 (0.08)	
	Google Fit	3125.7 (725.1)		3609.1 (822.5)		3836.3 (717.9)		1.80 (0.09)		1.28 (0.06)		1.71 (0.08)	
**Total activity count (counts per day)**													
	PuzzleWalk	343,351 (42,869)		363,626 (50,927)		321,134 (47,377)		3.42 (0.15)		0.96 (0.05)		1.52 (0.07)	
	Google Fit	256,596 (39,328)		288,175 (46,917)		304,998 (43,647)		3.42 (0.15)		0.96 (0.05)		1.52 (0.07)	
**Anxiety level**													
	PuzzleWalk	17.3 (2.1)		16.2 (2.2)		14.5 (2.9)		2.78 (0.13)		0.02 (0.00)		0.32 (0.02)	
	Google Fit	17.6 (2.0)		16.5 (2.0)		12.9 (2.6)		2.78 (0.13)		0.02 (0.00)		0.32 (0.02)	

^a^PA: physical activity.

^b^Estimated means (SE) for repeated-measures analysis of covariance when adjusted for age, sex, and BMI. ^c^*P*<.05, post hoc comparison within groups, between groups, or overall difference.

**Table 4:** Summary of changes in PA^a^ app use from the intervention start to the intervention end^b^.

Variable			Intervention start, mean (SE)		Eighth-week start, mean (SE)		Eighth-week end, mean (SE)		Time, *F* test (*η*_p_^2^; *df*=2,38)		Between-group, *F* test (*η*_p_^2^; *df*=2,38)		Time×group, *F* test (*η*_p_^2^; *df*=2,38)
**PA app use (minutes per day)**													
	PuzzleWalk	203.5 (62.6)^c^		82.9 (38.7)^c^		162.9 (48.3)^c^		0.80 (0.04)		4.37 (0.19)^c^		5.07 (0.21)^c^	
	Google Fit	12.9 (57.7)^c^		13.2 (35.7)		3.3 (44.5)^c^		0.80 (0.04)		4.37 (0.19)^c^		5.07 (0.21)^c^	

^a^PA: physical activity.

^b^Estimated means (SE) for repeated-measures analysis of covariance when adjusted for age, sex, and BMI. ^c^*P*<.05, post hoc comparison within groups, between groups, or overall difference.

**Table 5:** Correlations between PA^a^ and anxiety at baseline and their changes over time^b^.

Time point and assessments			Sedentary time (minutes per day)		Light PA (minutes per day)		MVPA^c^ (minutes per day)		Steps (steps per day)		TAC^d^ (counts per day)		Anxiety level
**Baseline**													
	Sedentary time (minutes per day)	1.00		−0.18		−0.46^e^		−0.53^e^		−0.49^e^		0.10	
	Light PA (minutes per day)	−0.18		1.00		0.20		0.17		0.33		0.11	
	MVPA (minutes per day)	−0.46^e^		0.20		1.00		0.93^f^		0.97^f^		−0.21	
	Steps (steps per day)	−0.53^f^		0.17		0.93^f^		1.00		0.92^f^		−0.21	
	TAC (counts per day)	−0.49^e^		0.33		0.97^f^		0.92^f^		1.00		−0.22	
	Anxiety level	0.10		−0.11		−0.21		−0.21		−0.22		1.00	
**Change**													
	Sedentary time (minutes per day)	1.00		−0.27		−0.59^f^		−0.66^f^		−0.54		−0.49^e^	
	Light PA (minutes per day)	−0.27		1.00		0.55^f^		0.50^e^		0.68^f^		0.41^e^	
	MVPA (minutes per day)	−0.59^f^		0.55^f^		1.00		0.91^f^		0.90^f^		0.40	
	Steps (steps per day)	−0.66^f^		0.50^e^		0.91^f^		1.00		0.87^f^		0.45^e^	
	TAC (counts per day)	−0.54^f^		0.68^f^		0.90^f^		0.87^f^		1.00		0.41^e^	
	Anxiety level	−0.49^e^		0.41^e^		0.40		0.45^e^		0.41^e^		1.00	

^a^PA: physical activity.

^b^Data are presented as Spearman correlation coefficients, *r*_s_.

^c^MVPA: moderate to vigorous physical activity.

^d^TAC: total activity count.

^e^*P*<.05.

^f^*P*<.01.

## Discussion

### Principal Findings

The findings from this feasibility study indicate that gamified PA-promoting mobile apps can be an effective tool for decreasing sedentary time and increasing MVPA in intellectually able adults with ASD. Notably, the PuzzleWalk mobile app, developed using BCTs and autism-specific design elements, was comparable with the commercially popular Google Fit in inducing changes in PA and sedentary time. However, the positive improvements in PA and sedentary time did not significantly reduce anxiety levels, although overall anxiety severity for participants with ASD was positively changed from moderate to mild at the end of the intervention.

It appears that intellectually able adults with ASD can benefit similarly from both commercially available and best practice–guided PA mobile apps. Although there were no significant differences in PA and sedentary time between the 2 groups, there were varying trends in the data according to groups, which are worthy of mention. In the PuzzleWalk group, there was a downward trend in sedentary time and a short-term upward trend in MVPA, step count, and total activity count. Conversely, there was a slight intervention-induced increase in light PA and a clear upward trend in steps and total activity counts in the Google Fit group. These observations, in conjunction with the significantly greater time spent on app use in the PuzzleWalk group compared with the Google Fit group, suggest that the specific research-based design elements of PuzzleWalk, including BCTs and competitive gamification, may pose an advantage over commercially popular apps that do not incorporate these elements. As shown in previous gamified PA interventions for neurotypical samples, adding competition elements to the intervention may be more effective in inducing PA or sedentary behavior changes than collaboration when there are no social connections among users [88,89]. It is of interest to see whether these trends are enhanced in a larger sample of adults with ASD.

Increasing evidence suggests that mobile app interventions can be effective in promoting PA mostly in the short term (eg, up to 3 months) [86]. In a recent study, the short- and long-term effects of a mobile phone–based PA app, together with brief in-person counseling, were evaluated for PA behavior change among inactive middle-aged women (n=210). The findings of this randomized clinical trial indicated that the intervention group’s MVPA and total steps were significantly increased during the first 3-month intervention period, although the intervention effect was not maintained in the following 6 months [88]. Similar results of regression fallacy have been reported in earlier randomized controlled trials that examined the efficacy of smartphone sensor–based interventions on PA promotion in healthy adults, which found a substantial PA decline during long-term follow-up [83,89]. Given that adherence and engagement in mobile app interventions generally decline before 3 months [33,86], the attenuation of intervention effects observed in this study (ie, regression of PA outcomes at the intervention end) may correspond with the overall decline in time spent on PA app use during the intervention period. As such, it is critical for mobile app interventions to maximize participant engagement with the app and exposure to the intervention through additional health behavior management strategies such as motivational short message services and telephone coaching [86,90].

This study aimed to identify an anxiety treatment adjunct to, and not in replacement of, conventional therapies. The overall anxiety level decreased from moderate to mild, with decreases in sedentary time and increases in PA over time, in both groups. Although this change was not statistically significant, it may be of clinical or personal value to adults with ASD. A recent review and meta-analysis reported that PA may have a protective effect against anxiety symptoms and disorders; however, the authors cautioned against drawing firm conclusions because of the limited and heterogenic available research [1]. Kim et al [91] recently reported that the optimal range of PA for decreasing anxiety symptoms was 600 to 9000 metabolic equivalents (METs) minutes per week and 1200 to 3000 METs minutes per week for neurotypical men and women, respectively [91]. Individuals with ASD in this study had mean PA levels of 569.9 (SD 45.6) METs minutes per week at the intervention end, which did not meet this criterion [92]; therefore, the intervention-induced changes in PA may have been insufficient to further affect anxiety levels. In addition, many adults with ASD have difficulty articulating their emotions and feelings, and this could have affected the ability of participants in this study to report the full extent of their anxiety symptoms [93]. Despite the lack of robust outcomes regarding intervention effectiveness, additional research on the long-term effects of PA interventions as a simple and economical way of alleviating the health burden of anxiety in adults with ASD is encouraged.

Interestingly, small intervention-induced changes in PA, sedentary time, and anxiety were unfavorably associated. Increases in light PA, steps, and total activity counts and decreases in sedentary time were associated with increased anxiety in adults with ASD. This is contrary to the preponderance of research and a general understanding of the relationships among PA, sedentary time, and anxiety [24,94]. Previous studies have shown that anxiety symptoms are associated with low PA levels and increased sedentary time [95]. Furthermore, Lee et al [96] found that adults with ASD reporting higher sedentary time had increased odds of developing physical and mental health conditions, including anxiety, associated with cardiovascular risk. The underlying mechanisms that explain the relationship between PA, sedentary time, and anxiety are understudied [1]; however, a potential explanation for the findings in this study is that PA-related social situations and environmental changes may have negatively contributed to anxiety symptoms in the sample [7,97]. Confusion and worries about social and communication situations were common anxiety triggers in adults with ASD in this study, as has been previously reported among those with ASD who are often self-aware of their incompetence and difficulty in social situations [7,65,97,98]. As the mobile apps used in this study prompted outdoor walking, which is difficult to perform in social isolation, it is reasonable to conclude that the prospect of entering the public to walk could have elevated anxiety in study participants. Specific types of anxiety were not assessed in this study; however, Hollocks et al [4] found that social phobia was a highly prevalent type of anxiety disorder in adults with ASD. Therefore, future efforts to study PA and sedentary time interventions for this population must consider the social expectations of the prescribed activity.

The major strengths of this study include (1) the use of a covariate-adaptive randomized controlled trial design to control the influence of covariates in the results [99], (2) objective assessment of PA and sedentary time, and (3) relatively good adherence to the study protocol and low attrition. Limitations include a small sample size; the use of self-report for the assessment of anxiety symptoms and app use time; and potential underestimation of PA, as accelerometry cannot accurately detect bicycling, swimming, and other upper-body movements [100]. Regardless, this feasibility, proof-of-concept study demonstrated that intellectually able adults with ASD could favor a gamified PA-promoting mobile app, and this use can induce small improvements in PA, sedentary time, and anxiety, which are worthy of further investigation.

### Conclusions

This study demonstrates that a gamified BCT-based mobile app, PuzzleWalk, may be able to decrease the level of sedentary time and create a short-term impact on increasing MVPA, daily steps, and total activity counts among adults with ASD. However, the findings also suggest that anxiety in adults with ASD was unfavorably related to increased light PA, steps, and total activity counts and decreased sedentary time after the intervention. Further longitudinal research is warranted to evaluate the long-term efficacy of PuzzleWalk in improving physical and mental health and to elucidate the underlying mechanisms that explain the roles of PA and sedentary time in changing anxiety symptoms in adults with ASD. Given the promising usability of a gamified app as a supplementary behavior change tactic, it is recommended that the design elements of mobile health interventions be user centered to meet the unique needs and leverage the strengths of the target population (eg, visual support for users with autism). Furthermore, as supported by many previous findings, mobile health interventions should focus on increasing sustainability and long-term behavior change that can continuously promote regular PA participation. Finally, there is a need for more experimental studies conducted in real-world settings to verify the evidence for gamification and other BCTs in underserved populations [62].

## Appendix

Multimedia Appendix 1 CONSORT-eHEALTH checklist (V 1.6.1).

## Abbreviations

ANCOVA: analysis of covariance
AQ-10: Autism Spectrum Quotient 10-item
ASD: autism spectrum disorder
BAI: Beck Anxiety Inventory
BCT: behavior change technique
DAA: daily anxiety assessment
ICC: intraclass correlation
MET: metabolic equivalent
MVPA: moderate to vigorous physical activity
PA: physical activity
